# Identification of Heavy Metals and Organic Micropollutants in Drinking Water Sources in Typical Villages and Towns in Northeast China

**DOI:** 10.3390/molecules27228033

**Published:** 2022-11-19

**Authors:** Guangyuan Wang, Jimin Shen, Shengyue Wei, Daxing Cai, Jinde Liu

**Affiliations:** 1 State Key Laboratory of Urban Water Resources and Environment, School of Environment, Harbin Institute of Technology, Harbin 150090, China; 2 Nanyang Traditional Chinese Medicine Development Bureau, Nanyang 473000, China; 3 Heilongjiang Hydrological and Water Resources Center, Harbin 150001, China

**Keywords:** northeast China, rural, drinking water sources, conventional water quality parameters, organic micropollutants

## Abstract

This study identified and detected the existence of major pollutants in northeast China. As an alpine region and an agricultural base, this region has representative significance in pollution research. We selected 56 samples from drinking water sources of typical villages and towns, focusing on the analysis of heavy metals and organic micropollutants in northeast China. The analysis results showed that Fe and Mn were the main metal elements exceeding the standard. The exceeding rates were 17.9% and 19.6%. Experiments showed that there were 19 kinds of pesticides, 6 kinds of OPEs, 2 kinds of PAEs, 22 kinds of PPCPs. The detection rate of these 49 kinds of organic micro-pollutants were 1.79~82.14%. The characteristics of organic pollution were extensive and varied. Many underground water samples had high level of micropollutants. The water quality parameters of drinking water sources in villages and towns showed close relation to local geological conditions and agricultural activities. Actions must be taken to control these parameters from the source of pollution.

## 1. Introduction

**Figure 1:**
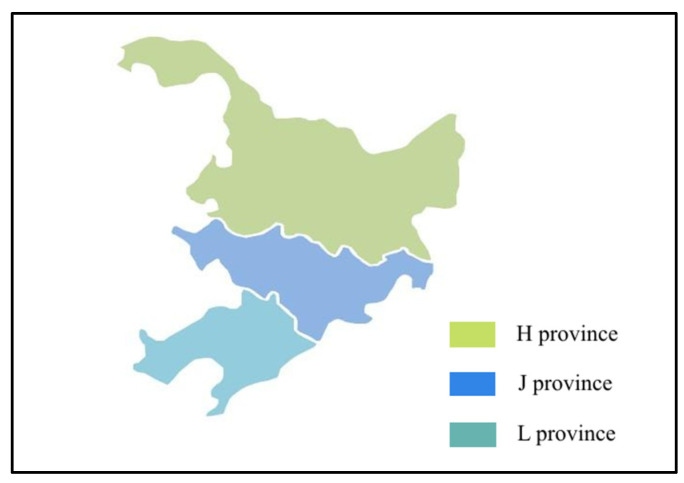
Administrative division of district M.

Northeast China is a typical alpine region, which contains H(Heilongjiang), J(Jilin), and L(Liaoning) provinces (Figure 1). It is also an important agricultural production base in China. The drinking water source of villages and towns is mainly surface water and shallow groundwater in this region. However, heavy metals and micropollutants jeopardize the safety of residents’ drinking water.

Human activities, such as the use of fertilizers and the discharge of domestic and industrial sewage, pollute natural water through surface runoff and underground leakage [1]. In fact, there are no suitable and efficient methods for village water treatment. Once the water is polluted, a high cost is paid to remove the pollutants for lack of conditions in villages. In order to reduce the pressure of village water treatment, we did this experiment to conclude the situation of pollution in northeast China and found possible reasons behind these phenomena so that we could stop the pollution from other processes. Thus, the aim of this study was to identify and analyze the existence and distribution characteristics of pollutants, trace the source of conventional pollutants, and provided statistics and theoretical support to guarantee water safety in villages and towns.

Heavy metals have been recognized as a hazard to human health [2]. This region was in trouble of dealing with a high level of Mn and Fe. Organic micropollutants had gained more and more attention because of their persistent existence in drinking water. The pollutants had strong polarity and stability which made it difficult for them to degrade naturally. The use of chemical fertilizers and pesticides caused a certain degree of non-point source pollution [3]. Exactly, micropollutants were widespread and existed in unexpected places. Laura M [4] et al. analyzed the water quality of 1204 underground water intake wells in the United States and found that 41% of the wells contained pesticide compounds, and a total of 109 pesticides and 116 species were found. PPCPs, as a type of micropollutant, appeared generally in surface water because of the extensive use of social drugs, cosmetics and shampoos [5]. For its widespread use, there were many sources of PPCPs. Yu [6] investigated the possible sources of PPCPs in the surface water of Qingdao and found that PPCPs even existed in treated water. 

**Figure 2:**
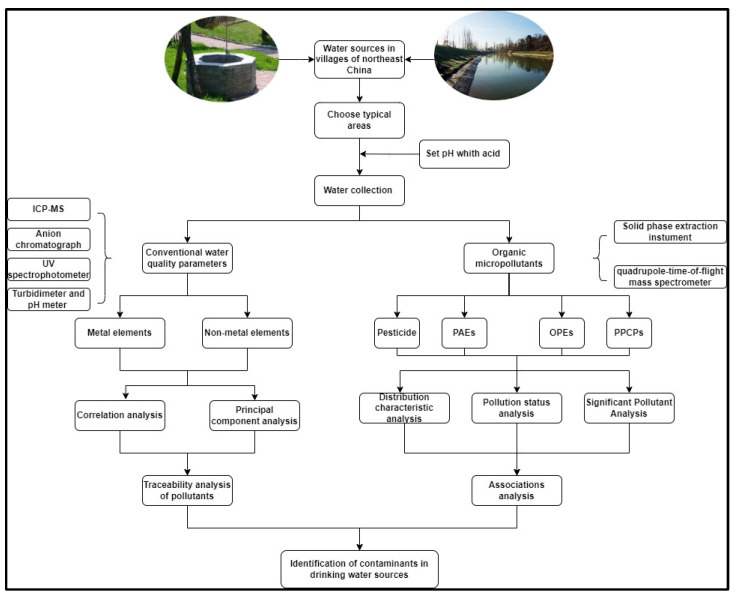
Flow chart of the whole experiment.

This severe situation required emergency measures to control the pollution in this area. This study collected drinking water samples from villages and towns in typical rice-growing areas, corn-growing areas, soybean-growing areas, alpine plains, and areas where agriculture, forestry, and animal husbandry intersected in northeast China. We evaluated eight heavy metals, four light metals, some non-metal parameters and thirty-nine organic micropollutants in four categories (Figure 2). We listed the pollutants and considered their damage to the water quality and human health. The identification and distribution of heavy metals and micropollutants provided a certain reference value.

## 2. Material and Methods

### 2.1. Selected Target Compounds and Chemicals

All chemicals were at least of analytical grade except as noted and used as received without further purification. All solutions were supplied by an ultrapure water system. Hydrochloric acid, potassium permanganate and concentrated sulfuric acid were purchased from J&K Scientific Co., Ltd. (Beijing, China). Sodium oxalate and Nessler’s reagent were purchased from Macklin Biochemical Technology Co., Ltd. (Shanghai, China). Methanol, Formic acid and Acetonitrile (all chromatography pure) from Sinopharm Chemical Reagent Co., Ltd. (Shanghai, China). C18(3 cc,500 mg) was purchased from Waters (Milford, CT, USA).

### 2.2. Study Site and Sample Collection

**Figure 3:**
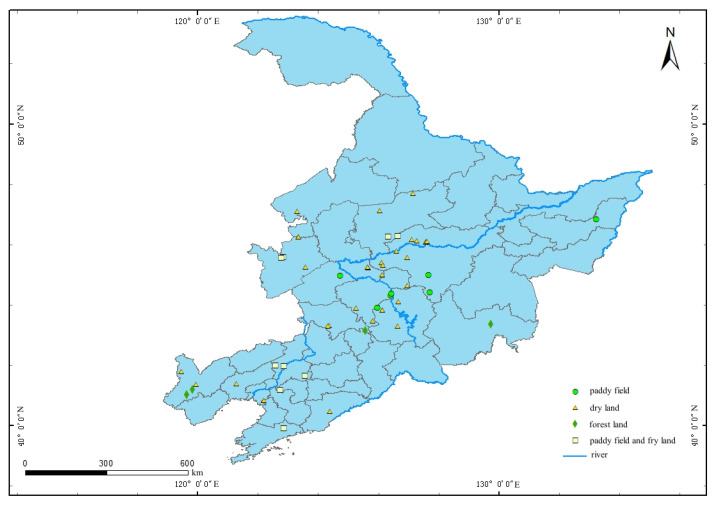
Sample points and division of regions.

In this study, we chose typical villages and towns in this region, such as rice planting and soybean and corn planting areas (Figure 3). The sample points covered 4 prefecture-level cities and 21 villages in H province, 6 prefecture-level cities and 19 villages in J province and 7 prefecture-level cities and 12 villages in L province. Additionally, sample points covered well water, river water, and lake water. It included 48 groundwater samples and 8 surface water samples. Among the underground water samples, there are 26 shallow underground water samples (5–50 m), which accounted for 46.4% of the total. The remaining 22 were deep underground water samples. Among the surface water samples, there were six reservoir water samples and two river water samples. To make the distribution more reliable, sufficient dispersion was ensured between the sample points. Therefore, the water samples were universal and comprehensive for the villages in northeast China. Apart from simply sampling, we recorded and collected the detailed information of geographic conditions of the sample points, the nearby hydrological environment, and possible pollution factors such as chemical mining industries. With this information, the sample points can be further analyzed after testing. All the samples were collected in glass bottles to avoid an unexpected reaction in the case that the properties of water changed. Some parameters like TDS and turbidity were detected immediately. After the detection, we set pH below 2 with sulfuric acid and nitric acid. The sample with sulfuric acid was for Cr detection. Then, the time and temperature of the water samples were recorded, and sent back to the laboratory within 48 h. During the transportation of water samples, ice packs were used to keep the water samples at low temperature (4 °C). The samples were stored in a dark place to inhibit the change of material components in the water and ensure the consistency of water quality parameters between sampling and testing. The collected samples were all extracted within 3 days and analyzed within half a month.

### 2.3. Sample Preparation

#### 2.3.1. Pretreatment for Metal Element Detection

For metal elements, the water samples were stored in glass bottles with a pH less than 2, and 5 ml of the water samples were passed through a 0.45 μm filter to remove the suspended solids or precipitates in the water samples. Digestion was performed to avoid high concentrations of organic matter present in the water sample from affecting the detection results.

#### 2.3.2. Pretreatment for Non-Metal Detection

For the determination of total organic carbon, two drops of 2 mol/L hydrochloric acid solution were added dropwise to pH less than 2 before sample loading to remove inorganic carbon in the water sample. For the determination of anions, all water samples are filtered with a 0.22 μm water-based filter membrane before the measurement to remove impurities in the water samples. Repeat the measurement twice for each water sample to obtain the average value. Other parameters can be detected by relevant instruments.

#### 2.3.3. Pretreatment for Micropollutants Detection

The residual concentration of organic micropollutants in drinking water is very low, especially the concentration of organic micropollutants in groundwater is mostly at the trace level of ng/L, and direct detection is often difficult. Therefore, it is necessary to enrich and concentrate water samples in advance [7]. The pretreatment method of the samples in this study selected solid-phase extraction technology. Compared with liquid–liquid extraction technology, solid-phase extraction technology has the advantages of less organic solvent usage, no emulsion extraction, and good method reproducibility and recovery rate [8]. At present, the commonly used solid-phase extraction cartridges in the laboratory mainly include silica-based cartridges represented by C18, polymer-based cartridges represented by HLB, and mixed-type strong cation cartridges represented by MCX. Comparing the enrichment and concentration effects of C18, MCX, and HLB solid-phase extraction cartridges on kinds of organic micropollutants in surface water, we chose the C18 cartridge to achieve the best enrichment effect and the highest recovery rate [9]. Therefore, in this experiment, C18 was selected as the filler for the solid phase extraction cartridge.

Pretreatment of water samples: First, use a 500 mL graduated cylinder to accurately measure 500 mL water samples, and filter them with a 0.45 μm filter to remove suspended particles in the water samples. The C18 solid phase extraction cartridge was activated with 10 mL of methanol and 10 mL of ultrapure water at a flow rate of 3 mL/min, and then 10 mL of ultrapure water was used to activate the cartridge. A C18 solid phase extraction cartridge was equilibrated at a flow rate of 3 mL/min. Concentrate 500 mL of water sample through a C18 solid phase extraction cartridge at a flow rate of 8 mL/min, and rinse with 10 mL of ultrapure water after the sample is loaded. Continue to extract under low vacuum for 30 min to ensure that the residual water in the C18 solid phase extraction cartridge is completely extracted. Finally, use 1 mL of methanol solution to elute at a flow rate of 1 mL/min, and accurately dilute the eluate to a 1 mL liquid phase vial for testing.

### 2.4. Instrument Analysis

#### 2.4.1. Inorganic Pollutant Detection

Metal elements were detected with an ICP-MS (Optima8300 from PerkinElmer, Waltham, MA, USA); turbidity was detected with a Turbidimeter (HACH-2100 Q from HACH, Loveland, CO, USA); TOC was detected with a total organic carbon analyzer (Multi N/C3100 from Analytik Jena AG, Jena, Germany); TDS was detected with a multi-parameter analyzer (HACH-sension378 from HACH, Loveland, CO, USA); ammonia nitrogen was detected with a UV spectrophotometer (DR-3900 from HACH, Loveland, CO, USA); anions were detected with a multifunctional high pressure anion chromatograph (URG9000 from Thermo Fisher Scientific Inc., Waltham, MA, USA); organic micropollutants were detected with a solid phase extraction instrument (USE-24 S from Thermo Fisher Scientific Inc., Waltham, MA, USA) and a quadrupole-time-of-flight mass spectrometer (G6546 Q-TOF LC/MS from Agilent Technologies).

#### 2.4.2. Organic Micropollutant Detection

In this experiment, we selected an Agilent liquid chromatography-G6546 quadrupole-time-of-flight mass spectrometer, which has the characteristics of a large, extended dynamic range and the benefit of high resolution. At the same time, combined with Agilent’s compound database and spectral library (PCDL), Mass Hunter software is used to perform seamless targeted screening of unknown substances. Under such conditions, thousands of organic micro-pollutants can be screened out through one injection.

Chromatographic conditions: chromatographic column EclipsePlus-C18 2.1 × 50 mm, 1.8 μm; flow rate 0.2 mL/min; column temperature 30 °C; injection volume 1 μL; mobile phase A was 0.02% formic acid aqueous solution, and mobile phase B was acetonitrile. The solution ratio in the mobile phase was controlled by a gradient method, with a stop time of 40 min and a post-run time of 1 min. The mobile phase gradient is shown in Table 1.

Mass spectrometry conditions: ion source was ESI with Agilent Jet Stream Technology, positive ion mode; drying gas temperature 325 °C, drying gas flow rate 6 L/min; sheath gas temperature 350 °C, sheath gas flow rate 11 L/min; capillary voltage 4000 V; nozzle voltage 500 V; fragmentation voltage 110 V; mass-to-charge ratio range of mass-to-charge ratio acquisition in primary mass spectrometry mode 50–1050 m/z; scan rate 1 spectrum/second; mass scanning range and acquisition speed of secondary mass spectrometry mode were the same as primary mass spectrometry; MS mode collision energies 0, 10, 20, 40 V; reference ions m/z 121.0508 (M+H)^+^ and 922.0097 (M+H)^+^.

Optimization of mobile phase selection: Because the tested organic micro-pollutants are all carried out in the form of positive ions, in theory, most of the precursor ions are formed by adding H^+^, Na^+^ and NH4^+^. Thus, we tried adding appropriate formic acid to the mobile phase to improve the ability of the mobile phase to elute compounds. Therefore, methanol-water, methanol-0.02% formic acid aqueous solution, acetonitrile-water and acetonitrile-0.02% formic acid aqueous solution were selected as mobile phases in this experiment to compare the separation effects of different components relative to organic micropollutants in natural water. It was found that when acetonitrile-0.02% formic acid aqueous solution was used as the mobile phase, the chromatographic peak separation effect was the best, and the response value of most organic micro-pollutants was relatively high. Therefore, acetonitrile-0.02% formic acid aqueous solution was selected as the mobile phase in this experiment. 

Non-target identification of organic micropollutants in water samples: when using high-resolution mass spectrometry to identify compounds, an accurate precursor ion and a secondary fragment ion must be required, and the mass deviation of the precursor ion should also meet the requirements. Under the preset chromatographic and mass spectrometry conditions, select the primary mass spectrometry mode to run the water sample after solid phase extraction to obtain primary mass spectrometry raw data. The primary mass spectrometry raw data was analyzed by Qualitative Workflows (B.08.00) qualitative software, the mass deviation of the precursor ion was set to 10 ppm. The batch non-target identification of organic micro-pollutants was carried out in combination with PCDL, and the results of the library search and comparison were filtered out. The overall score for organic micropollutants above 70 was chosen. We input the mass-to-charge ratio (m/z) and retention time of the preliminarily screened organic micropollutants into the Targeted List interface in Targeted MS/MS and set the retention time deviation to 0.32 min and the quadrupole resolution to medium (~4 m/z), and screened the secondary fragment ion data for the preliminary screened organic micropollutants. Finally, we compared the secondary mass spectrometry data with the secondary mass spectrum of the organic micropollutants in the library. When the mass deviation of the secondary fragment ions of the organic micropollutants was less than 5 ppm, it could be confirmed that the compound was the target organic micropollutant. 

## 3. Results and Discussion

### 3.1. Occurrence of Heavy Metals

#### 3.1.1. Occurrence of Manganese Ions 

**Figure 4:**
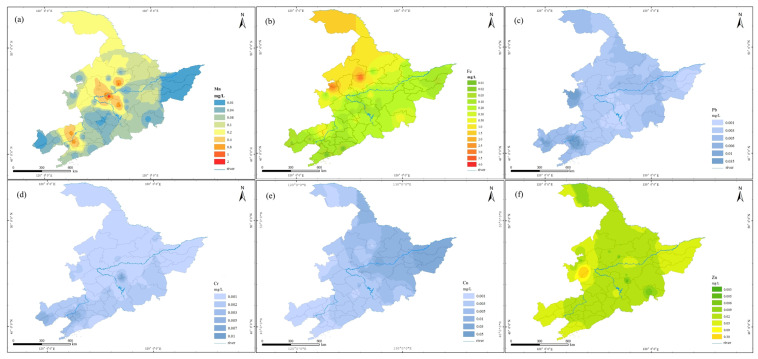
Prediction map of concentration distribution of heavy metals in drinking water sources in typical villages and towns in M area (**a**) Mn, (**b**) Fe, (**c**) Pb, (**d**) Cr^6+^, (**e**) Cu and (**f**) Zn.

**Figure 5:**
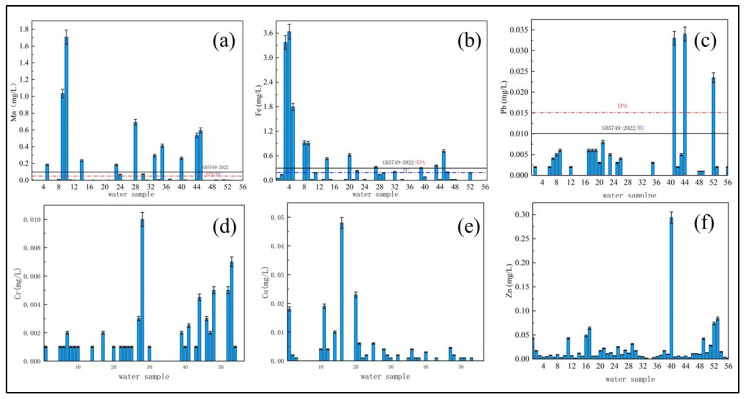
Distribution of heavy metal concentrations in drinking water sources in typical villages and towns in M area: (**a**) Mn, (**b**) Fe, (**c**) Pb, (**d**) Cr, (**e**) Cu and(**f**) Zn.

The standard of Mn in drinking water was 0.1 mg/L (according to the new regulation “GB5749–2022”). Considering that the drinking water in villages and towns in M area was mainly groundwater, the distribution of Mn accounted for a considerable proportion of water resources safety. According to the Chinese geological environmental survey, it was found that Mn in groundwater in China was the most important heavy metal pollutant, especially in the M area, and that the phenomenon of manganese exceeding the standard was the most serious [10]. After sampling and testing, it could be seen that among the 56 typical village water samples in M area, the qualified rate of the metal Mn index was the lowest among all metal indexes, only 80.4%, and the number exceeding the standard was as high as 11. From the overall distribution of Mn concentration (Figure 4a), the Mn content in most areas was between 0.08 and 0.2 mg/L. The detection range was 0~1.706 mg/L (Figure 5a), and the average value was 0.114 mg/L, which was greater than the limit of drinking water. According to the drinking water standard of the United States (EPA) and the European Union (EU), another two samples exceeded the standard, and the pass rate dropped to 76.8%. The Mn index in drinking water sources in rural areas of J province was relatively good, being in the range of 0.04–0.08 mg/L. The content of Mn in the central part of L province was relatively high, ranging from 0.1–0.8 mg/L. The phenomenon of excessive Mn in H province was the most serious.

#### 3.1.2. Occurrence of Iron Ions

The Fe standard for drinking water was 0.3 mg/L. The detection range of the sample parameters was 0~3.64 mg/L (Figure 5b), and the average value was 0.27 mg/L. There were 10 points that exceeded the drinking water limit, and the maximum exceeded the standard by 12 times. The qualified rate of the indicator was only 82.1%. This excess of Fe in drinking water would cause chronic poisoning, nerve damage and other diseases [11], so the phenomenon of excessive Fe should not be underestimated. Some studies have shown that the phenomenon of excessive Fe in groundwater in the M region was relatively serious. From 2013 to 2017, the annual Fe excess rate in L province was 40%, while the Fe excess rate in J province was between 18.18% and 72.73%. The average annual rate of excess was 60% [10]. 

From the spatial distribution of Fe (Figure 4b), the phenomenon of Fe exceeding the standard in H province was relatively serious, with an average of 0.56 mg/L, slightly higher than the limit of drinking water. The excessive points were mainly in the underground drinking water sources which lay to the south of City B and the junction between the east and the City of S. The concentration was about 1–3 mg/L. The average Fe in Province L was 0.09 mg/L. In the villages and towns in the west, it was about 0.5~1 mg/L.

#### 3.1.3. Occurrence of Lead Ions

The drinking water standard for Pb is 0.01 mg/L, and the detection range of the sample index was 0~0.003 mg/L (Figure 5c). As a heavy metal with a high usage rate in the industry, the irregular discharge in industrial areas was often the main reason for high Pb levels. There was often Pb pollution in paddy fields. Soluble Pb in the soil can enter the water body through precipitation leaching and leakage. Long-term drinking damages the normal growth and development of the body [12]. Among them, there were three sampling points where Pb exceeded the standard, the maximum value was 0.034 mg/L, and the average value was 0.004 mg/L, which was far lower than the limit of drinking water, and the pass rate of the index was 94.6%. According to the spatial distribution (Figure 4c), the Pb concentration in the source water of villages and towns in this area was 0–0.003 mg/L, and the overall situation was relatively good. Excess points were mainly concentrated in the central area of L Province and the western area of J Province, and the exceeding points were underground water with a well depth of 10–15 m, most likely because the Cr in the soil migrated into the water body. At the same time, some studies have shown that the scale in natural water body had a certain effect on the leaching of Pb in the pipeline, and the change of water quality might lead to the increase of the solubility of the scale, thereby releasing the Pb in the pipeline into the water [13]. Knowies [14] et al. found that Al in the water might affect the precipitation of Pb in the pipeline. Under the condition of low alkalinity, the Pb leaching experiment was carried out on the water pipe containing Pb, and it was found that the content of Pb released in the pipeline was related to the water body. The residual Al content was highly positively correlated with the light metal content in the water source.

#### 3.1.4. Occurrence of Chromium Ions

The drinking water standard of Cr is 0.05 mg/L. The detection range was 0~0.01 mg/L (Figure 5d), and the average value was less than 0.001 mg/L, which was far lower than the world’s major drinking water safety standards, indicating that in the M region. There was no Cr pollution in the drinking water sources of villages and towns. Hexavalent Cr pollution in groundwater not only exists in industrially polluted areas, but also in non-industrially polluted areas due to the over-exploitation of groundwater, which may lead to a funnel area in the groundwater level, causing the migration and diffusion of Cr and causing Cr pollution [15]. However, from the perspective of the spatial distribution of Cr (Figure 4d), the areas with high Cr concentration were mainly concentrated in L province. The reason might be the excessive exploitation of groundwater here, resulting in the formation of the underground funnel area, thus creating conditions for the migration of Cr, which led to the phenomenon of pollution diffusion. At present, the groundwater exploitation rate of main cities in L province had reached 68.97~88.11%, which directly or indirectly led to the formation of two large funnel areas in the L province’s groundwater [16].

#### 3.1.5. Occurrence of Copper and Zinc Ions

The drinking water standard of Cu and Zn is 1.0 mg/L. According to the spatial distribution of the two elements (Figure 4e,f), there was no problem of Cu and Zn contamination in this area, and the detection range of Cu was 0~0.048 mg/L (Figure 5e), with an average value of 0.001 mg/L; the maximum detected value of Zn was 0.29 mg/L (Figure 5f), and the average content was 0.003 mg/L. The relative content of Zn index in drinking-water source water in the eastern and western parts of H province, the western part of J province and the western part of L province was high, between 0.05 and 0.30 mg/L, but they all met the drinking-water quality requirements.

#### 3.1.6. Occurrence of Cadmium and Barium Ions

The drinking-water standard of Cd is 0.005 mg/L, and Ba is 0.7 mg/L. Compared with other metal elements, the content of Cd and Ba in the water source was low. Cd cannot be detected in 80.4% of the sample points, while Ba appeared in all samples. Although the rate of Ba was high and all the points of use were involved, the detection range was only 0.008~0.309 mg/L, which was far lower than the standard limit. Therefore, the impact of Cd and Ba on the water resources of villages and towns in the M area was quite limited at present, and it was not necessary to control it.

In summary, it can be seen that Fe and Mn pollution in this area was currently serious, and the problem of Mn had constituted a huge threat to the drinking water of villages and towns. However, although other heavy metal elements such as Cr, Cu and Zn were involved in a wide range of regions, their pollution levels were generally mild and did not pose a threat to the safety of drinking water in villages and towns. Therefore, for the drinking water of villages and towns, only by adopting practical Fe and Mn removal technology can the safety of drinking water be more comprehensively covered in the villages and towns.

Two main factors were considered to be possible reasons for the high content of this condition. On the one hand, M region was the heavy industry base of the whole country. Production activities in heavy industry inevitably involved heavy metal elements, which represented a higher emission of water polluted by Fe and Mn. Under this condition, when the dry season came, the pollution would be more serious because the discharge was constant. Thus, the contents of Fe and Mn would present seasonal changes. On the other hand, it was the geological factor that influenced the pollution. In northeast China, there were rock layers and ores rich in Fe and Mn. The landform, sedimentary environment and groundwater runoff of this area led to more leaching of Fe and Mn from the layers. Iron and manganese ores were mostly associated with minerals. With the effect of weathering, ores were washed into water bodies with water flow in flood season, polluting surface water. Some of them even penetrated soil and flew into groundwater, causing serious heavy metal pollution.

### 3.2. Occurrence of Light Metals

#### 3.2.1. Occurrence of Sodium and Potassium Ions

**Figure 6:**
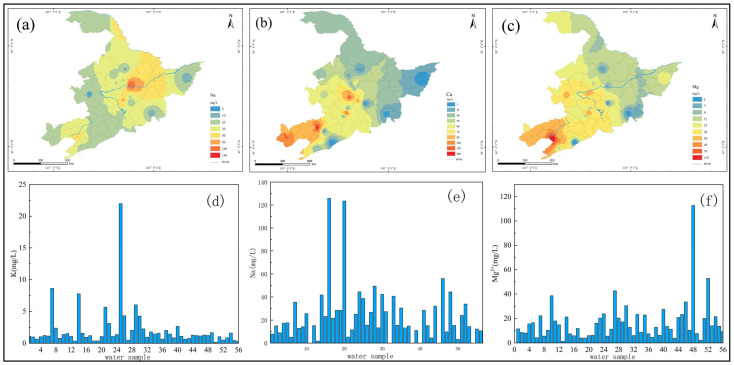
(**a**–**c**) are the predicted map of Na^+^, Ca^2+^ and Mg^2+^ concentration distribution of drinking water sources in M area; (**d**–**f**) are concentrations of Na^+^, K^+^ and Mg^2+^ in M area.

Sodium and potassium are essential trace elements for the human body, also known as nutrients. Sodium and potassium are widely distributed in soil and groundwater, and their content is affected by factors such as runoff conditions, geological conditions, and ion exchange adsorption [17]. From the distribution prediction result (Figure 6a), it can be seen that the Na^+^ index in the drinking water source water of villages and towns in this area was better than the drinking water limit of 200 mg/L. However, the Na^+^ content in drinking water source water in different regions varies greatly: the maximum detected value was 125.8 mg/L, the minimum value was not detected, and the range was relatively large. The average concentration of Na^+^ in the drinking-water source water of villages and towns here was 11.46 mg/L. The average Na^+^ content in the drinking-water source water of villages and towns in H province was the highest in the three provinces of 26.66 mg/L, and the lowest in L province was 19.67 mg/L. From the potassium ion distribution map (Figure 6d), it can be seen that the maximum K^+^ concentration in the M area was 22 mg/L, the minimum was 0.175 mg/L, and the mean value was 0.381 mg/L. From the perspective of spatial distribution, the concentration of K^+^ in the drinking water sources of villages and towns in J province was relatively high, with an average of 2.86 mg/L; the lowest was in L province, with an average of 0.99 mg/L.

#### 3.2.2. Occurrence of Calcium and Magnesium Ions

Epidemiological studies have shown that when calcium (Ca^2+^) and magnesium (Mg^2+^) intakes are appropriate, the higher the intake, the lower the risk of cardiovascular disease and hypertension, and the lower the mortality rate from cancer. However, when the content of calcium and magnesium ions was high, it will seriously affect the taste of water [18]. From the Ca^2+^ concentration map of drinking water source sampling points, it can be seen that the maximum Ca^2+^ concentration of drinking water source sampling points in typical villages and towns was 209.7 mg/L, the minimum value was 7.7 mg/L, and the mean value was 65.6 mg/L.

From the prediction result of Ca^2+^ concentration distribution (Figure 6b), it can be seen from the spatial distribution of Ca^2+^ concentration that the Ca^2+^ concentration in drinking water sources of villages and towns in H province was generally 40–50 mg/L, with an average value of 48.2 mg/L; J The concentration of Ca^2+^ in drinking water sources of villages and towns in the province was generally 60–70 mg/L, with an average value of 68.26 mg/L; the concentration of Ca^2+^ in drinking water sources of villages and towns in L province was relatively high at 70–100 mg/L, with an average value of 90.81 mg/L. The concentration of Ca^2+^ greater than 100 mg/L was mainly concentrated in the villages and towns in the western and northern regions of L province.

From the Mg^2+^ concentration map at the sampling point (Figure 6f), it can be seen that the detection range of Mg^2+^ in the sampling points of drinking water sources in typical villages and towns was 1.3 mg/L~112.7 mg/L, and the detection range was large. The value was 11.6 mg/L. From the prediction result of Mg^2+^ concentration distribution (Figure 6c), it can be seen that the spatial distribution of Mg^2+^ concentration was very different. The overall concentration of Mg^2+^ in the drinking water sources of typical villages and towns in H province was 9–15 mg/L, with an average value of 11.2 mg/L, which was slightly lower than the average value of the overall Mg^2+^ concentration in region M. With an average value of 15.9 mg/L, the overall concentration of Mg^2+^ in drinking water sources in villages and towns in L province was relatively high, especially in the southwest of L province, where the Mg^2+^ concentration ranges from 70 to 115 mg/L.

### 3.3. Conventional and Inorganic Non-Metallic Parameters

#### 3.3.1. Sensory and Physical Index Analysis

The relevant parameters measured in this study mainly included pH, conductivity, turbidity, hardness and total dissolved solids. The pH of drinking water sources in typical villages and towns in Region M all met the standard, which was within the range of 6.5 to 8.5 required for domestic drinking water, with an average value of 7.27.

Conductivity refers to the electrical conductivity of liquid expressed by a numerical value, which is closely related to the mineral content in the water. Natural water generally contains few inorganic salts, and the conductivity ranges from 50 to 1500 µS/cm. In this study, the detection range of electrical conductivity in typical villages and towns of M region was between 80.4 and 1475 µS/cm with an average value of 581.5 µS/cm. From the perspective of spatial distribution, the electrical conductivity of drinking water sources in L province is as high as 773.9 µS/cm, and the lowest in H province was 445.6 µS/cm, which was lower than the average electrical conductivity of drinking water sources in typical villages and towns of this region.

**Figure 7:**
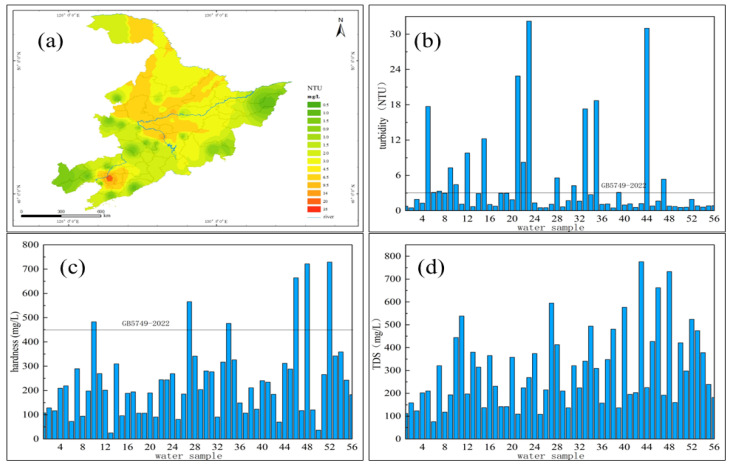
Distribution and concentrations of turbidity, hardness and total dissolved solids in M area. (**a**,**b**) turbidity (**c**) hardness (**d**) TDS.

Turbidity is an important criterion for evaluating water quality, and the sanitary standard for drinking water (GB5749–2022) stipulates that its limit is 3 NTU. It could be seen from the figure (Figure 7a,b) that the detected minimum value was 0.48 NTU, the maximum value was 32.2 NTU, and the average value was 4.6 NTU above the limit. Turbidity was the smallest standard rate among all the conventional parameters of drinking water sources in typical villages and towns. The number of exceeding the standard was 17, and the pass rate was 69.6%. It could be seen that turbidity was an important factor affecting the quality of drinking water sources in M area. From the perspective of the spatial distribution of turbidity, the turbidity index of drinking water source water in villages and towns in H province was not ideal. The turbidity in large areas of the province exceeded the standard, and the turbidity index was generally between 3 and 7 NTU. As the temperature in the M area decreased, low-temperature and low-turbidity water would also be formed, which would increase the difficulty of water treatment. 

It could be seen from the figure (Figure 7c) that the average hardness of drinking water sources in this area was 237.3 mg/L, which was less than the limit of 450 mg/L for drinking water. The detection range was 24.9–728.9 mg/L, and the pass rate was 89.3%; there were six sampling points whose hardness exceeded the standard, and all the exceeding points were groundwater. The areas with higher hardness were mainly concentrated in the groundwater of villages and towns in the southwestern region of L province. The hardness of the water source water in these areas was generally between 650 and 750 mg/L. The hardness is between 450 and 550 mg/L. The overall distribution of hardness in village water source water in H province is between 120 and 400 mg/L with an average value of 179 mg/L. The situation was relatively good. The overall distribution of hardness in village water source water in J province is between 300 and 400 mg/L. There were several possible reasons for the high level of hardness. First, sewage treatment was not up to standard. Acid, alkali and salt in surface sewage entered the soil layer, and through chemical processes such as combination decomposition and ion exchange, Ca^2+^ and Mg^2+^ in the soil were dissolved, making the hardness high. Second, the content of organic matter in the water body was higher than other regions. In that case, a higher concentration of carbon dioxide was produced through the degradation of water microorganisms, which changed the carbon balance of the original water body and promoted the dissolution of calcium carbonate. In addition to the high hardness caused by the nature of the water, the acid rain would also lead to the decrease of the pH of the water and then increase the dissolution of Ca^2+^ and Mg^2+^. The combination of various effects caused the high hardness in the western part of L-province.

The total dissolved solid concentration of the water sampling points in typical villages and towns of M area all meet the standard (Figure 7d), which were all less than the drinking water limit of 1000 mg/L. The detection range is 75.8–776 mg/L, and the average value was 301.7 mg/L. It was generally believed that while the content of total dissolved solids was less than 600 mg/L, the water quality tastes better. The total dissolved solid concentration in H province was generally between 155 and 275 mg/L, and the total dissolved solid concentration in the southern part of H province was higher. The areas with higher total dissolved solid concentration in L province were mainly concentrated in the southwest. The total dissolved solid concentration in drinking water source in the eastern part of J province was higher, between 650 and 780 mg/L.

#### 3.3.2. Inorganic Non-Metal Parameters

The inorganic non-metallic parameters measured in this study included F^−^, Cl^−^, SO_4_^2−^, TN and TP. Fluorine is a trace element that plays an important role in growth and bone metabolism. Appropriate intake of fluoride is beneficial to the human body, but excessive intake of fluoride will cause fluorosis, and lack of fluoride will cause various physiological and pathological changes. The limit value is 1.0 mg/L [19].

**Figure 8:**
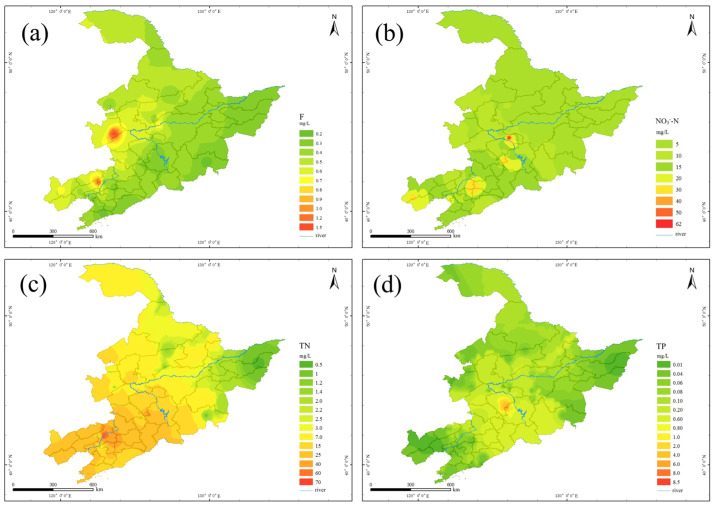
Prediction of Concentration Distribution of Inorganic Nonmetals in Drinking Water Sources in Typical Villages and Towns of Region M: (**a**) F, (**b**) NO_3_^—^N, (**c**) (TN) and (**d**) (TP).

There were only 2 samples exceeding standards; the maximum concentration was 1.41 mg/L, the minimum was 0.12 mg/L, the average was 0.43 mg/L, and the compliance rate was 96.4%. From the spatial distribution of F^−^ concentration (Figure 8a), the F^−^ concentration of drinking water sources was generally between 0.2 and 0.5 mg/L. The excessive F^−^ concentration points were mainly concentrated in the eastern part of J province. The concentration was between 1.0 and 1.5 mg/L, which was slightly higher than the limit of drinking water.

**Figure 9:**
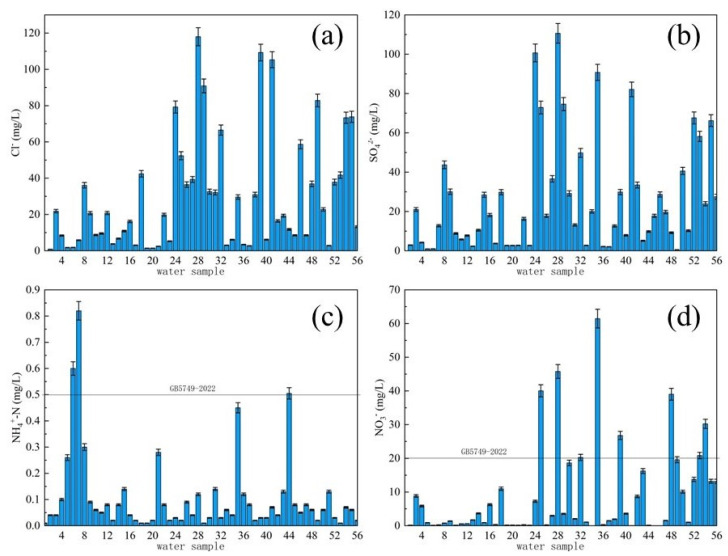
Inorganic ion concentration distribution of drinking water sources in typical villages and towns of M area: (**a**) l^−^, (**b**) SO_4_^2−^, (**c**) NH_4_^+^-N, (**d**) NO_3_^−^-N.

According to the Cl^−^ concentration map of drinking water source sampling points in typical villages and towns (Figure 9a), the detection range of Cl^−^ concentration was 0.85~118.01 mg/L, which were all lower than the limit of 250 mg/L required for drinking water. From the perspective of the spatial distribution of Cl^−^ concentration, the average Cl^−^ concentration was 28.75 mg/L. The Cl^−^ concentration in the drinking water sources of villages and towns in province J was relatively high among the three provinces with an average of 42.46 mg/L, and the lowest in province H was 11.36 mg/L.

It could be seen from the SO_4_^2−^ concentration map (Figure 9b) of the sampling points of drinking water sources that the maximum value of SO_4_^2−^ detected in typical village drinking water sources was 110.64 mg/L, and the minimum value was 0.57 mg/L with an average value of 25.85 mg/L, which was less than the limit of 250 mg/L for drinking water. The spatial distribution of SO_4_^2−^ concentration in different regions varied greatly. The overall concentration of SO_4_^2−^ in H province was 10–20 mg/L, with an average value of 11.77 mg/L. In L province, the areas with relatively high concentrations were mainly concentrated in the western region with an average concentration of 29.58 mg/L.

Ammonia nitrogen (NH_4_^+^-N) is a relatively common and serious drinking water pollutant. Ammonia nitrogen is a nutrient in water that can lead to the phenomenon of eutrophication of water. It is the main oxygen consumption pollutant in water, which is toxic to fish and some aquatic organisms [20]. From the NH_4_^+^-N concentration map (Figure 9c) of typical village drinking water source, it could be seen that the detection range of NH_4_^+^-N concentration at the sampling point was 0.01~0.82 mg/L, and there were three points of village drinking water sources. The concentration of NH_4_^+^-N in the water exceeded the drinking water limit of 0.5 mg/L, and the compliance rate of NH_4_^+^-N was 94.6%.

According to the NO_3_^−^-N concentration map (Figure 9d) of drinking water source sampling points, the NO_3_^−^-N concentration at different points was quite different, and the detection ranged from 0 mg/L to 61 mg/L, and the maximum value exceeded the standard by three times with an average value of 8.3 mg/L. Eight sampling points had NO_3_^−^-N concentrations exceeding the drinking water limit of 20 mg/L, and the types of water sources exceeding the standard were groundwater. From its distribution prediction result (Figure 8b), it could be seen that the spatial distribution of NO_3_^−^-N varied greatly. The concentration of NO_3_^−^-N in drinking water sources of villages and towns in H province was roughly between 5 and 20 mg/L. The concentration of NO_3_^−^-N in L province was roughly between 15 and 20 mg/L with an average value of 12.75 mg/L. The areas with higher NO_3_^−^-N concentrations ranged from 40 to 65 mg/L, exceeding the standard by 2 to 3.2 times.

The total nitrogen (TN) is mainly NH4^+^-N and NO_3_-N, and also includes some organic nitrogen. The detection range of TN in the sampling points of drinking water sources was 0.41–69.3 mg/L with an average value of 10.73 mg/L. From the prediction map of TN concentration distribution (Figure 8c), it could be seen that the spatial distribution of TN concentration in M area was extremely uneven, and the TN concentration in L province was relatively high. The average concentrations of TN in H, J and L provinces were 2.4 mg/L, 14.4 mg/L and 18.91 mg/L, respectively. The areas with high TN concentration were mainly concentrated in J and L provinces. The concentration in the center of L province was as high as about 70 mg/L.

The amount of total phosphorus (TP) is also a major factor in evaluating water quality. The detection range of TP was 0~8.48 mg/L, and the detection rate was 62.3%. Phosphorus was not detected in 20 samples, and only one sampling point had a higher total phosphorus content of 8.48 mg/L, indicating that M area is free from phosphorus pollution. According to the prediction result of TP concentration distribution (Figure 8d), it could be seen that the total phosphorus in the drinking water sources of villages and towns in H and L provinces was generally distributed between 0 and 0.1 mg/L, and the total phosphorus in J province was in the range of 0–0.1 mg/L. 

### 3.4. Total Organic Pollutants Analysis

**Figure 10:**
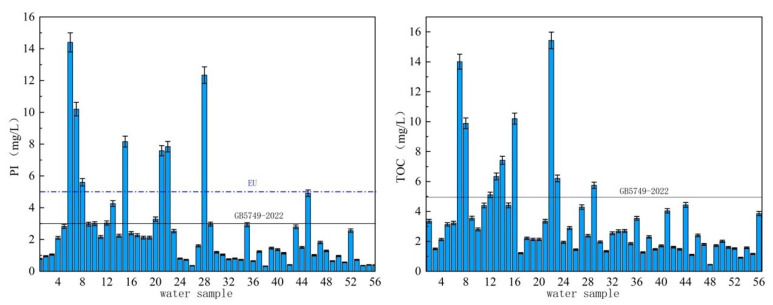
Oxygen consumption and TOC graph.

Oxygen consumption is a major parameter to measure organic matter in water. Studies have shown that oxygen consumption is positively correlated with sensory properties of water, disinfection by-products and carcinogenicity of water. As can be seen from Figure 10, there were 11 sampling points with PI exceeding the drinking water limit of 3 mg/L which had a maximum value of 14.4 mg/L. The qualified rate was 80.4%, and the detection range was 0.32–14.4 mg/L. The type of water source exceeding the standard was mainly surface water, accounting for 72.2%, indicating that surface water in villages and towns in this area was more susceptible to organic pollution than groundwater, and the source of surface water pollution was more complex. From the perspective of spatial distribution, the average PI of drinking water sources was 2.6 mg/L, which was slightly lower than the limit for drinking water. The average PI of drinking water sources in villages and towns in H, J and L provinces were 4.2 mg/L, 1.8 mg/L and 1.3 mg/L, respectively. The areas with excessive oxygen consumption in H province were about 5~15 mg/L, while PI of underground water sources in L province was relatively high with an overall distribution of 3 to 6 mg/L.

Total organic carbon (TOC) refers to the total amount of organic matter in water reflected by carbon content. The larger its value, the higher the concentration of organic matter. There are 9 areas where the TOC concentration of drinking water source sampling points in villages and towns exceeds 5 mg/L (Figure 10). Among them, 6 of samples were surface water, indicating that the surface water is highly contaminated with organic matter. The detection range of TOC was 0.4–15.4 mg/L with an average value of 3.4 mg/L. From the perspective of spatial distribution, the TOC concentration in the surface water sources in H province was relatively high, ranging from 6 to 15 mg/L. The TOC concentration in J province had an average of 2.55 mg/L. 

### 3.5. Occurrence of Organic Micropollutants

In this study, more than 2100 organic micropollutants were screened in 48 samples of groundwater and 8 samples of surface water in 52 typical villages and towns in M region. Forty-nine organic micropollutants were detected, including 19 pesticides, 6 organophosphates (OPEs), 2 phthalates (PAEs), and 22 pharmaceutical and personal care product (PPCPs) pollutants.

#### 3.5.1. Pesticide Detection

Only 1% of the applied amounts of pesticides can be completely absorbed by crops during use, and the remaining 99% of pesticides will remain in the soil, water and atmosphere. Polar and highly soluble pesticides especially can easily enter through surface runoff and leaching into water, but their concentration in natural water is often less than 1 μg/L [21]. The pesticides tested for this time included 11 herbicides, 4 insecticides and 4 fungicides.

The detection rates of 11 herbicides and their degradation products are as follows: atrazine (76.79%), desethylatrazine (42.86%), 2-hydroxyatrazine (35.71%), Deisopropylatrazine (14.29%), Permethazine (12.50%), Metolachlor (12.50%), Nicosulfuron (10.71%), Promethazine (8.93%), Atrazine pass (5.36%), imazapyr (3.57%) and sicameth (1.79%).

Herbicide residues were not detected in only 3 of the 56 water samples. The other 53 water samples were contaminated with at least one herbicide. Nearly four-fifths of the water samples where herbicides were detected contained degradation products, especially atrazine. Atrazine and its degradation products were not detected in only 4 water samples, and at least one atrazine or its degradation products was detected in the remaining 52 water samples. The detection rate of atrazine was as high as 76.79%, and the detection rate of deethylated atrazine was also as high as 42.86%. This was due to the relatively high degradation conversion rate of herbicides in the natural environment. Especially atrazine, deethylated atrazine and alachlor are more mobile in the environment; these herbicides were more difficult for colloids to absorb, and were often transported to water bodies when large rainfall climates occurred and they degraded under natural conditions [22]. Therefore, the detection rate of herbicide degradation products in natural water bodies would be higher, but the concentration was usually only a few ng/L to several hundred ng/L [23].

**Figure 11:**
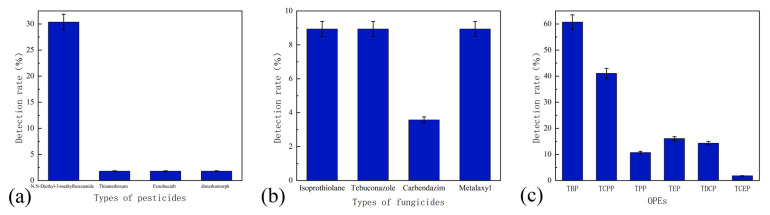
Detection map of organic micropollutants: (**a**) pesticides, (**b**) fungicides and (**c**) OPEs.

Four insecticides were detected (Figure 11a) which were DEET, thiamethoxam, sec-butacarb and dimethomorph. DEET was the most commonly detected insecticide, with a detection rate of 30.36%. Thiamethoxam, secbucarb and dimethomorph were all detected in only one water sample. DEET is a widely used insect repellant that had repellent effects on a variety of insects and is used by approximately 200 million people worldwide [24]. Although many studies had proved that DEET was the safest and most effective broad-spectrum insect repellent, the human body still could not be exposed to DEET for a long time, and products containing DEET would penetrate into the blood after contacting the skin and serious adverse skin reactions such as blisters and erosions occur [25].

A total of four fungicides were detected: rice blast, tebuconazole, carbendazim and metalaxyl. It could be clearly seen that the detection rate of fungicides was relatively small, less than 10% (Figure 11b). These four fungicides were systemic fungicides, with higher water solubility than general fungicides, and could easily enter the water body, thereby affecting the quality of the water environment [26].

Province H, Province J and Province L all had one site where no pesticide residues were detected, which were sites No. 1, No. 34 and No. 51, respectively. The high detection rate of pesticide residues indicated that pesticide compounds had entered the source water of villages and towns in M area through surface runoff or infiltration. Among the three pesticides, herbicides had the highest detection rate, followed by insecticides and fungicides.

A total of 10 herbicides were detected in H province, but only 1 herbicide, atrazine, was not detected; 8 herbicides were detected in J province, and 3 herbicides were detected in L province, all of which were atrazine and its degradation products. Among the herbicides in the three northeastern provinces, the highest detection rate of atrazine was more than 50%, and the detection rate of atrazine in H province was as high as 95.45%, which was related to the widespread use of atrazine in the rural areas of H province.

Among the 56 water samples, fungicides were detected in 11 water samples, and 4 fungicides were detected in H province. The highest detection rate of rice blast spirit is 18.18%. Three kinds of fungicides, but no carbendazim was detected in the water samples of J province, and the detection rates of the other three fungicides ranged from 4.76% to 9.52%. Rice blast spirit and carbendazim were not detected in water samples from L province, and the highest detection rate of metalaxyl was 15.38%.

Only one insecticide, DEET, was detected in the sampling points in H province, and its detection rate was as high as 31.82%, indicating that DEET was the main insecticide used in villages and towns in H province. Two kinds of insecticides, DEET and dimorpholine, were detected in J province. DEET was also the main insecticide used in villages and towns in J province, with a detection rate of 38.10%. The detection rate of pesticides in L province was 69.23%, the highest in the three northeastern provinces, in which dimethomorph was not detected, and the detection rates of the other three pesticides ranged from 7.69% to 15.38%.

#### 3.5.2. PAEs Detection

Two PAEs were found: bis(2-ethylhexyl) phthalate DHXP (67.86%) and diethyl phthalate DEP (26.79%). PAEs are widely used as plasticizers in plastic products such as food packaging materials, medical devices, toys and gloves; in addition they are added to paints, cosmetics and adhesives [27].

Since PAEs are not added to the polymer in the form of covalent bonds, they can easily enter the environment, and the phenomenon of random dumping of plastic waste in rural areas is common, causing PAEs to enter the water body and affect the water quality [28]. Both PAEs are listed as priority pollutants by the US EPA. DHXP is often used as an unconventional parameter of water quality in China, with a limit of 0.008 mg/L, while DEP is used as only a reference indicator for water quality with a limit of 0.3 mg/L.

Two kinds of PAEs were both detected in Region M, and the detection rates of DHXP in Province H and Province J were both greater than 80%, indicating that DHXP was the main phthalate compound in the water source water in the villages and towns of these two provinces. DHXP (15.38%) and DEP (15.38%) were detected in four sites in L province.

#### 3.5.3. OPEs Detection

Six OPEs were detected: tributyl phosphate (TBP), tris(1-chloro-2-propyl) phosphate (TCPP), triethyl phosphate (TEP), tris(1,3-diphosphate) chloropropyl) ester (TDCP), triphenyl phosphate (TPP) and tris(2-chloroethyl) phosphate (TCEP) (Figure 11c).

OPEs are not added to substances by chemical bonding, and OPEs are semi-volatile substances, which can easily enter the environment through exudation and volatilization, and enter water bodies through processes such as precipitation and sedimentation [29]. OPEs have been detected in sewage, surface water, groundwater and even drinking water, and the concentration was often around several hundred ng/L [30]. Among the six OPEs, the detection rates of TBP and TCPP were 60.71% and 41.07%, respectively, and the detection rate of TCEP was the lowest, only 1.79%. This might be due to the fact that the ban of TCEP in the past two decades had resulted in a significant decrease in the concentration of TCEP in water bodies, while TCPP, as its substitute, had a higher detection rate and concentration in various water bodies [31].

All six OPEs were detected in J province, and the overall detection rate was 95.24%. Among them, TBP and TCPP were the main OPEs in the water source water of villages and towns in J province, with the detection rate ranging from 42.86% to 57.14%. Four kinds of OPEs were detected in L province and H province, respectively. TBP was the main OPEs in villages and towns in L and H provinces, and the detection rates were 53.85% and 68.18%, respectively. 

#### 3.5.4. PPCPs Detection

Although the content of PPCPs in many water bodies is very low (ng/L~μg/L) and does not cause acute poisoning [32], due to its persistence and strong stability in the environment, there is an unforeseen possibility of the harm of the water, and the consequent problem of the complex components after water treatment is the control and reduction of disinfection by-products.

A total of 22 PPCPs were detected, of which 16 were pharmaceuticals, 1 cosmetic, and 5 other PPCPs. Drug varieties included 3 analgesics, 3 antidepressants, 6 antibiotics, 1 diet pill, 1 beta blocker, 1 anesthetic, and 1 progestin. The detection rates of 22 PPCPs are shown in Table 2. The PPCPs with the highest detection rate were oleic acid amide, which was detected in 46 water samples. Oleic acid amide has a wide range of uses and is often used as a sleeping pill and sedative. The detection rates of dibutyl adipate, amantadine and L-phenylalanine were also relatively high, ranging from 16.07% to 19.64%. Paracetamol, sulfa antibiotics, metoprolol, nicotine and caffeine were all detected, but the detection rate was low at 1.79%.

PPCPs such as caffeine, nicotine, paracetamol and metoprolol had low removal rates in WWTPs and were often used as parameters of wastewater from WWTPs, so the presence of these substances could be considered as source water contaminated by raw wastewater [33,34]. Sulfonamide antibiotics were widely used in veterinary medicine. 30–90% of sulfonamide antibiotics were excreted through feces, and sulfonamide antibiotics were highly water-soluble, which led to their weak affinity for soil and was easily transferred from soil to water. Therefore, sulfonamide antibiotics were often used as parameters of agricultural pollution in water bodies [35,36]. The detection of these PPCPs also indicated an important factor in the pollution of water source water in villages and towns caused by sewage leakage.

There were many types of PPCPs detected in M region, among which 13 species were detected in the H province, 11 species were detected in the J province, and 6 species were detected in the L province. Oleic acid amide was the PPCPs with the highest detection rate of water species in this region, and the detection rates in the three provinces were 86.38%, 85.71%, and 69.23%, respectively. The detection rate of dibutyl adipate in province H was also as high as 40.91%. PPCPs were all detected in the 22 water samples in H province, indicating that the water source water in H province was seriously polluted by PPCPs. No PPCPs were detected at points 34, 35, and 37 in J province, with an overall detection rate of 85.71%. There were two sites in L province where PPCPs are not detected.

#### 3.5.5. Distribution Analysis of Organic Micropollutants

**Figure 12:**
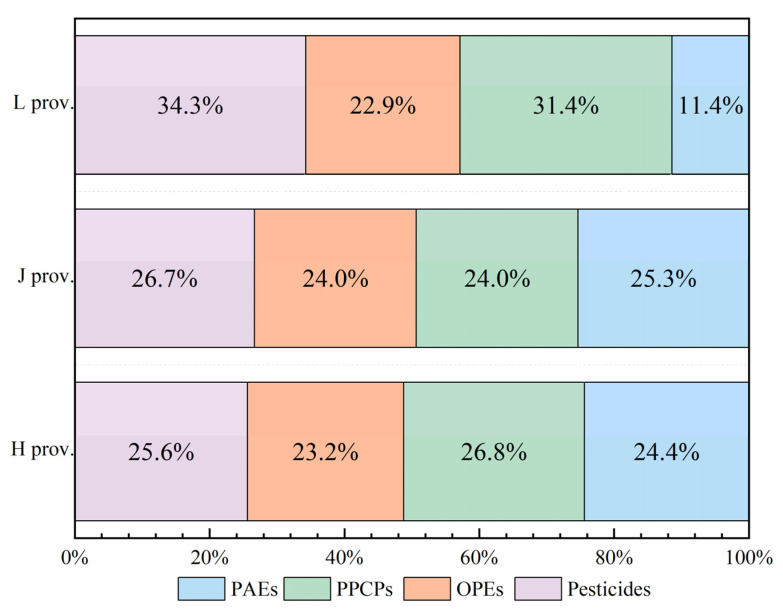
Relative composition of organic micropollutants in different regions.

The distribution of organic micro-pollutants reflected the production and life of the region to a certain extent. From the relative composition diagram of organic micropollutants in different regions, it could be seen that pesticides and PPCPs were the main organic micro-pollutants in the water source water of villages and towns in M region. The proportion was between 50.7% and 65.7%, and the distribution of the four organic micropollutants in the water source water in the villages and towns of the H and J provinces was relatively uniform, indicating that the water source water in the villages and towns of the H and J provinces was polluted by these four organic micropollutants. The main pollutants in water source water of L province were pesticides, accounting for 34.3% (Figure 12), followed by PPCPs, which also accounted for more than 30%. The proportion of PAEs was small, indicating that the water source water in villages and towns in L province was relatively less polluted by PAEs.

**Figure 13:**
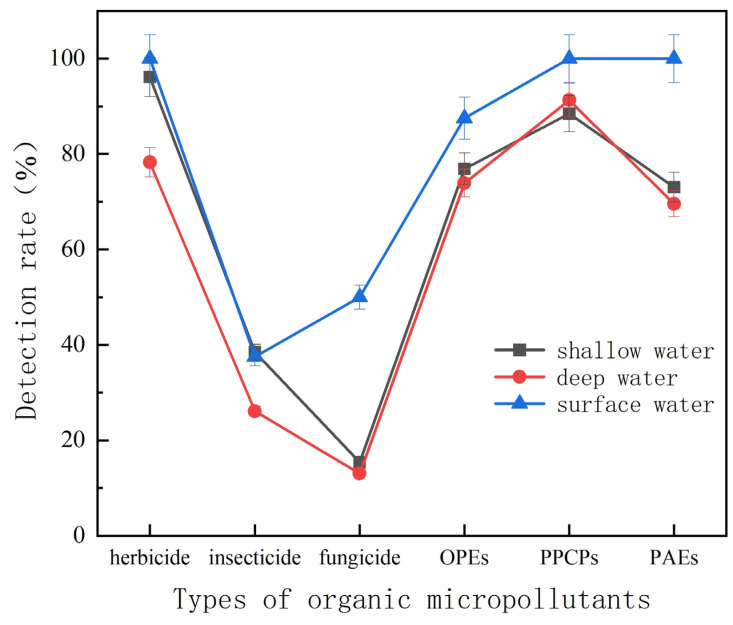
Detection of organic micropollutants in different water sources.

In fact, in addition to differences in spatial distribution, organic micropollutants in the same area also had certain differences due to different water sources. The detection rate of organic micropollutants in different water sources (Figure 13) showed that the detection rates of these six organic micropollutants in surface water were significantly higher than those in groundwater, especially the detection rates of herbicides, PPCPs and PAEs. The rate was as high as 100%. Twenty kinds of organic micro-pollutants were detected in No. 15 surface water source, and 12 organic micro-pollutants were detected in each surface water sample on average. The high detection rate of organic micropollutants in surface water sources in villages and towns also reflected that surface water sources were more susceptible to agricultural point and non-point source pollution. More than 10 organic pollutants were detected at points 2, 5, 17, and 27 in groundwater sources, and the detection rate of herbicides and pesticides was also similar to that of surface water, indicating that herbicides and pesticides had a higher possibility to infiltrate underground water sources through soil leakage and underground runoff than other four types of organic pollutants.

The distribution characteristics of the detection rate of herbicides in different water sources were surface water > shallow water well > deep water well. The detection rate of herbicides in deep groundwater was also as high as 78.26%, of which atrazine and its degradation products contributed the most. Atrazine and desethylatrazine were also detected in the 200 m deep well at points 38 and 54. Ren J et al. also detected atrazine and its degradation products in the groundwater of 130 m and 380 m in the rural area of Zhangjiakou [37]. This fully showed that due to the extensive use of atrazine in villages and towns, coupled with its greater polarity and strong hydrophilic properties, atrazine and its degradation products had been transferred through seepage into deep groundwater, which posed a threat to the quality of groundwater. Once groundwater is polluted on a large scale, its treatment conditions are more severe than surface water, the cost is higher, the technical requirements are more limited, and the available treatment methods are limited. Insecticides were detected in both surface water and groundwater, and DEET had the highest detection rate in both surface water and groundwater sources. The distribution characteristics of the detection rate of fungicides in different water sources were surface water > shallow water well > deep water well. The detection rate of fungicides in surface water was significantly higher than that in groundwater, indicating that fungicides were less able to enter into groundwater sources. The residues of fungicides in surface water were mainly rice blast spirit, while in groundwater were mainly metalaxyl and tebuconazole. Carbendazim was not detected in groundwater sources.

The distribution characteristics of the detection rate of OPEs in different water sources were surface water > shallow water well > deep water well. The main residues of OPEs in surface water were TBP, and the detection rate was 75%. The detection rate of TBP and TCPP in groundwater sources was between 37% and 50%. The distribution characteristics of the detection rate of PPCPs in different water sources showed that surface water > groundwater. The detection rate of PPCPs in deep water wells was slightly higher than that in shallow water wells. The detection rate of PPCPs in surface water was 100%. The main residues of PPCPs were oleic acid amides, and the groundwater sources were also mainly oleic acid amides.

The distribution characteristics of the detection rate of PAEs in different water sources were surface water > shallow water well > deep water well. The detection rate of PAEs in groundwater was significantly lower than that in surface water. The PAEs residues in the surface water were mainly DHXP, and the groundwater was also mainly DHXP, and the detection rate of DEP was 25%.

With the detection of these parameters, we formed a list (Table 3) of micropollutants which required effective treatment in real water supply. More attention was paid to control the content of these pollutants. It represented general regularity of pollution in villages of alpine regions.

## 4. Conclusions

In this study, we preliminarily demonstrated the regional distribution of water quality parameters and established screening methods for different pollutants. We found that their distribution characteristics indicated a tie association with local agriculture and geology. Heavy metals especially Mn and Fe had become big threats to water quality. Generally, the most widespread organic pollutant is atrazine, which makes viable and low-cost treatments of atrazine in villages a priority. Research showed a significant migration so far though varieties of routes. Different land types expressed different rates of migration. Further study should be done to clarify the certain reasons and indicators and to stop the spread of the pollution before it is too late.

At present, it is necessary to combine water treatments with the current situation of the region and comprehensively consider the causes and transfer methods of the pollution. Then, we can attach great importance to the source and process and establish a more reasonable detection mechanism to reduce the pressure on the treat end. Through consolidating the statistics, we could avoid the ineffectiveness and low performance of a single data point. With these methods, more difficulties would be solved in the safety of drinking water in villages and towns.

## Figures and Tables

**Table 1:** Mobile phase gradient.

Time/Min	Mobile Phase A/%	Mobile Phase B/%	Maximum Pressure Limit/Bar
0	95	5	1000
3	95	5	1000
5	90	10	1000
16	80	20	1000
22	70	30	1000
28	40	60	1000
34	5	95	1000
36	5	95	1000
36.01	95	5	1000
40	95	5	1000

**Table 2:** PPCPs Detection Rate.

Number	Category	Name	Detection Rate
1	antibiotic	Amantadine	17.86%
2		Lincomycin	3.57%
3		L-Tyrosine	1.79%
4		8-Hydroxyquinoline	1.79%
5		Sulfadimethylisopyrimidine	1.79%
6		Sulfamethazine	1.79%
7	Analgesics	Antipyrine	7.14%
8		paracetamol	1.79%
9		Dihydrocodeine	1.79%
10	antidepressant	L-Phenylalanine	16.07%
11		Sulpiride	1.79%
12		Diazepam	1.79%
13	cosmetic	Dibutyl adipate	19.64%
14	diet pills	mazindol	3.57%
15	beta-blockers	Metoprolol	1.79%
16	Anesthetic	Lidocaine	1.79%
17	progesterone	Progesterone	1.79%
18	other	Oleamide	82.14%
19		nicotine	8.93%
20		melamine	5.36%
21		caffeine	1.79%
22		5-Methylbenzotriazole	1.79%

**Table 3:** The list of organic pollutants required to be controlled.

Number	Category	Name	CAS Number
1	pesticide	Atrazine	1912–24-9
2		Metolachlor	51218–45-2
3		Propazine	139–40-2
4		Nicosulfuron	1119910–09-4
5		Diethyltoluamide	134–62-3
6		Thiamethoxam	153719–23-4
7		Isoprothiolane	50512–35-1
8		Metalaxyl	57837–19-1
9	OPES	TBP	126–73-8
10		TCPP	13674–84-5
11		TPP	115–86-6
12		TEP	78–40-0
13		TDCP	13674–87-8
14	PPCPs	Oleamide	301–02-0
15		Dibutyl adipate	105–99-7
16		Amantadine	768–94-5
17		Phenylalanine	63–91-2
18		Antipyrine	60–80-0
19		Acetaminophen	103–90-2
20		Sulfamethazine	57–68-1
21	PAEs	DHXP	117–81-7
22		DEP	84–66-2

## Data Availability

All data generated or analyzed during this study are included in this article. There are no separate or additional files.

## Sample Availability

Samples of the compounds are not available from the authors.
